# Scoring German Alternate Uses Items Applying Large Language Models

**DOI:** 10.3390/jintelligence13060064

**Published:** 2025-05-29

**Authors:** Janika Saretzki, Thomas Knopf, Boris Forthmann, Benjamin Goecke, Ann-Kathrin Jaggy, Mathias Benedek, Selina Weiss

**Affiliations:** 1 Department of Psychology, University of Graz, 8010 Graz, Austria; mathias.benedek@uni-graz.at; 2 Department of Psychology, Charlotte Fresenius Hochschule München, 80797 Munich, Germany; 3 Department of Psychology, LMU Munich, 80802 Munich, Germany; 4 GESIS—Leibniz Institute for the Social Sciences, 68159 Mannheim, Germany; thomas.knopf@gesis.org; 5 Institute of Psychology, University of Münster, 48149 Münster, Germany; boris.forthmann@uni-muenster.de; 6 Hector Research Institute of Education Sciences and Psychology, University of Tübingen, 72072 Tübingen, Germany; benjamin.goecke@uni-tuebingen.de (B.G.); ann-kathrin.jaggy@uni-tuebingen.de (A.-K.J.); 7 Institute for Psychology, University of Hildesheim, 31141 Hildesheim, Germany

**Keywords:** creativity, divergent thinking, assessment, automated scoring, large language models, alternate uses task, German, GPT

## Abstract

The alternate uses task (AUT) is the most popular measure when it comes to the assessment of creative potential. Since their implementation, AUT responses have been rated by humans, which is a laborious task and requires considerable resources. Large language models (LLMs) have shown promising performance in automatically scoring AUT responses in English as well as in other languages, but it is not clear which method works best for German data. Therefore, we investigated the performance of different LLMs for the automated scoring of German AUT responses. We compiled German data across five research groups including ~50,000 responses for 15 different alternate uses objects from eight lab and online survey studies (including ~2300 participants) to examine generalizability across datasets and assessment conditions. Following a pre-registered analysis plan, we compared the performance of two fine-tuned, multilingual LLM-based approaches [Cross-Lingual Alternate Uses Scoring (CLAUS) and the Open Creativity Scoring with Artificial Intelligence (OCSAI)] with the Generative Pre-trained Transformer (GPT-4) in scoring (a) the original German AUT responses and (b) the responses translated to English. We found that the LLM-based scorings were substantially correlated with human ratings, with higher relationships for OCSAI followed by GPT-4 and CLAUS. Response translation, however, had no consistent positive effect. We discuss the generalizability of the results across different items and studies and derive recommendations and future directions.

## 1. Introduction

Responses in, scores out. No recruiting and training of human raters. No waiting for weeks of coding. How does that sound? Researchers interested in creative thinking have long dreamed of a psychometrically sound automated solution to score the creative quality of responses in divergent thinking (DT) tests. While early attempts of automated scoring yielded promising results (for an overview, see Forthmann and Doebler 2022; Paulus and Renzuli 1968), significant advancements have been made only recently with the advent of large language models (LLMs; Goecke et al. 2024; Organisciak et al. 2023; Patterson et al. 2024a; Zielińska et al. 2023). To date, most automated scoring approaches have been developed and tested using English-language data, particularly in prototypical DT tasks such as the alternate uses task (AUT; Guilford 1967; Wallach and Kogan 1965; Wilson et al. 1954). However, recent studies have begun to explore the feasibility of applying automated scoring methods to other languages, either by first translating responses into English or by making use of their increasing multilingual capacities (Forthmann and Doebler 2022; Patterson et al. 2024a; Stevenson et al. 2022; Zielińska et al. 2023). However, little is known about the specific conditions under which automated scoring performs best in languages such as German. Therefore, this work investigates the performance of three LLM-based approaches for creativity scoring of German AUT responses.

### 1.1. Assessment of Divergent Thinking

Individual differences in creative thinking ability are typically assessed using DT tasks, which require participants to generate creative ideas in response to open-ended questions (Guilford 1950; Torrance 1972). For example, in the highly popular AUT, participants are asked to come up with creative uses for everyday objects (e.g., a brick) within limited time (Saretzki et al. 2024a). Various approaches for scoring the creative quality of DT responses have been proposed, such as coding responses based on a test manual, computing their sample-based infrequency (e.g., uniqueness scoring), or evaluating them by human raters consistent with the Consensual Assessment Technique (Amabile 1982, 1996; Baer et al. 2004). The rater-based method offers several advantages, as it allows raters to simultaneously consider multiple dimensions of creative quality and enables a robust handling of unique responses that may not be covered by test manuals. However, this rater-based approach also comes with notable downsides. First, human judgements are inherently subjective (Mouchiroud and Lubart 2001; Silvia et al. 2008). Rater agreement can be influenced by individual characteristics and rating conditions—for instance, experienced raters tend to achieve higher inter-rater reliability (Ceh et al. 2022), while cognitive workload can lead to greater disagreement (Forthmann et al. 2017b). Additionally, rating consistency depends on the specific rating instructions, which often vary across research labs. Second, human ratings are time-consuming and costly. Studies employing multiple DT tasks easily yield a few thousands of responses, depending on factors such as sample size, task instructions (e.g., focusing on the quality or quantity of responses), and time on task. As a result, ratings of a larger pack of responses can delay the research process and become expensive (Forthmann et al. 2023). Therefore, an effective automated scoring system for DT tasks across different languages would greatly enhance the reproducibility and efficiency of creativity research.

### 1.2. Automated Scoring of Divergent Thinking Tasks

Early computational approaches to evaluating creativity date back to text-mining methods, which laid the foundations for more advanced techniques incorporating natural language processing. First attempts relied on linguistic variables such as elaboration (i.e., word count) as predictors for the creativity of ideas (Paulus and Renzuli 1968). Later, methods shifted focus toward assessing originality by measuring the semantic distance estimated based on co-occurrences within large text corpora, mathematically represented as vectors in high-dimensional spaces (Landauer et al. 1998)—between a response and the problem cue.

Several studies have demonstrated positive relationships between semantic distance scores and human creativity ratings, but correlations across responses were modest (Beaty and Johnson 2021; Dumas et al. 2021; Yu et al. 2023). For example, semantic distance scores were compared across data from 12 languages and correlations were found to range from *r* = 0.23 (for Hebrew) to *r* = 0.52 (for English; German: *r* = 0.41; Patterson et al. 2023).

Earlier automated scoring methods relying on semantic distance and word counts have recently been surpassed by supervised machine learning and Transformer Language Models (TLMs) for evaluating creative responses (Organisciak et al. 2023). Supervised learning involves training models on labeled datasets, enabling them to make predictions on new, unseen data. These models often leverage decoder-only transformer architectures—such as those used in Generative Pre-trained Transformer (GPT) models—and can be fine-tuned to better align with human evaluations, thereby achieving high correlations with human ratings and outperforming base models (Organisciak et al. 2023; Zielińska et al. 2023). Recent studies report that fine-tuned supervised models can reach response-level correlations of up to 0.70 or even 0.80 with human ratings, with even higher correlations for scores aggregated across responses and when considering latent correlations (DiStefano et al. 2024; Goecke et al. 2024; Luchini et al. 2025b). These correlations lead to the assumption that supervised models may perform similarly to human raters in creativity scoring—maybe even more consistently and certainly much faster.

Even though ratings by fine-tuned models show high correlations with human ratings for held-out data, comparisons of the performance for completely new study data remain scarce. Moreover, while fine-tuned multilingual models are rapidly advancing, empirical evidence on their performance across different languages is still rare. One study found that the Open Creativity Scoring with Artificial Intelligence (OCSAI), based on GPT-3, worked well for Polish AUT data, achieving response-level correlations ranging from *r* = 0.42 to *r* = 0.72. Notably, translating responses into English before scoring did not yield any substantial improvements (Zielińska et al. 2023). Similarly, another study showed that a fine-tuned multilingual LLM reliably predicted the originality of stories across 11 languages in the held-out data (*r* > 0.70), including German (r = 0.75; Luchini et al. 2025a). These results suggest that LLM-based scoring approaches are increasingly capable of handling non-English data with high accuracy. However, empirical evidence remains limited regarding how well these models generalize to new datasets, particularly for German AUT responses.

### 1.3. The Present Research

While previous studies have demonstrated the potential of LLM-based approaches for the automated scoring of creativity, research on their effectiveness in non-English languages remains limited. In particular, little is known about how well these models perform when scoring prototypical DT tasks like the AUT in languages like German. Additionally, it remains an open question whether translating responses from the target language into English before scoring improves performance.

The present study addresses this existing research gap by systematically examining the performance of three LLM-based approaches for automatically scoring German AUT responses. Specifically, we investigated how different LLM architectures align with human ratings and whether scoring original German responses differs from scoring responses that have been automatically translated into English beforehand.

To this end, we focused on LLMs specifically trained for the creativity context as well as a general-purpose model. We compared two fine-tuned models—the Cross-Lingual Alternate Uses Scoring (CLAUS) and OCSAI—which have been explicitly trained on DT responses, with the base version of GPT-4, a versatile but non-specialized model. The main pre-registered research questions (RQs) were as follows (see pre-registration at: https://aspredicted.org/b8tx-xs3p.pdf, accessed on 13 July 2024):

RQ1: How do creative evaluations by LLMs compare to human raters for German AUT responses?

RQ2: Which LLM method performs best?

RQ3: Are findings generalizable across datasets and items?

We expected substantial positive correlations between LLMs and human ratings of AUT responses (Organisciak et al. 2023) but had no specific expectations regarding the relative performance of specific LLMs under specific language conditions.

## 2. Materials and Methods

### 2.1. Transparency and Openness

All accompanying materials, data, and analysis scripts are publicly accessible in an Open Science Framework (OSF) repository: https://osf.io/eaqtb/, accessed on 5 September 2024. All deviations from the protocol are reported below.

### 2.2. Data Sources

We compiled published as well as unpublished rated German AUT data across five research groups. The eight lab and online studies included 49,391 responses from 2320 participants for 15 different alternate uses objects.^1^ Studies varied in sample characteristics (e.g., age, educational level, etc.) as well as in certain task characteristics such as task instructions, items, and timing but all studies involved human creativity ratings of AUT responses. Key characteristics of the included studies are presented in Table 1. A more detailed overview of study-specific characteristics—including task instructions, items used, timing, and the scored DT dimensions—is available on the OSF.

### 2.3. Human Creativity Ratings

In all datasets, individual AUT responses were coded by at least two human raters for creativity on a 5-point scale, which was homogenized for all studies to the range of 1–5. In each study, raters underwent initial training where they were instructed on how to evaluate responses. While the definition of what characterized a creative response varied slightly across studies, it generally emphasized both its novelty (in the sense of being unusual, original, unique, or surprising), and effectiveness (in the sense of being appropriate, useful, clever, interesting, exciting, or humorous). Inter-rater reliabilities ranged from substantial (Study 1: ICC(3,3) = 0.786, 95% CI: [0.779, 0.793]) to almost perfect agreement (Study 8: ICC(3,3) = 0.902, 95% CI: [0.890, 0.913]) at the study level (see Table 1). The exact rater guides used are available as Supplemental Material.

### 2.4. Large Language Model Creativity Ratings

Three LLM architectures were considered in this study: CLAUS, OCSAI, and GPT-4.^2^

CLAUS employs a fine-tuned version of the XLM-RoBERTa language model to predict human creativity ratings for AUT responses (Patterson et al. 2024a). It was developed and validated using AUT responses from 12 languages (including German) and is available as part of the Creativity Assessment Platform (CAP; https://cap.ist.psu.edu/claus, accessed on 19 September 2024), an open-access website for the administration and automated scoring of creativity tasks in verbal, visual, science, technology, engineering, and mathematics domains. After uploading the AUT prompt and response data as a .csv file to CLAUS, it returns creativity scores in the range between 0 and 1 (uncreative to highly creative), with a precision of up to 10 decimal places. Multilingual OCSAI (version used: 1.6), developed by Organisciak et al. (2023; https://openscoring.du.edu/scoringllm, accessed on 17 December 2024), employs a GPT-4o-mini based network trained on a dataset of 27,000 human-rated AUT responses. It provides scores from 1 to 5, with higher scores reflecting greater originality. GPT-4, developed by OpenAI, represents a transformer-based LLM trained through self-supervised learning on a large corpus of text. Its architecture allows it to generate context-sensitive responses by modeling text probabilities.

While CLAUS and OCSAI do not require explicit prompting in their scoring process, GPT-4 was prompted in a way that reflected the essential instructions of the human raters across the eight studies, highlighting the relevance of both novelty and effectiveness of responses, but without giving specific example data (i.e., zero-shot). The instructions asked to provide differentiated ratings from 10 to 50 to make them more comparable to the rating range of the other LLMs as well as the average human ratings:

*Evaluate how creative the following use for the object %s is: %s.*

*An idea should be considered creative if it meets the following criteria: On the one hand, a creative idea should be novel (in the sense of being unusual, original, unique, or surprising), and on the other hand, it should also be effective (in the sense of being appropriate, useful, clever, interesting, exciting, or humorous).*

*Rate each idea on a scale from 10 (not creative at all) to 50 (very creative).*

The symbols “%s” indicate placeholders that were automatically replaced by the object and response from the input table for each individual prompt. However, it is important to note that CLAUS does apply an implicit prompt in the background, which is also included in the exported file (e.g., *Identify how surprising, creative, unexpected, or interesting the following alternate use for the object %s is: %s.*). Similarly, OCSAI employs a structured few-shot text-to-text prompt, as described in Organisciak et al. (2023).

Data collection via GPT-4 was conducted between 29 October and 20 November 2024.^3^

### 2.5. Alternate Uses Task Data Preparation

Human ratings of the AUT data were transformed to a five-point scale [from 1 (*not at all creative*) to 5 (*very creative*)] if not originally used, scaling linearly between the minimum and maximum score. Invalid responses that were flagged by different exclusion codes across labs were uniformly set to zero. Zeros were not included in the mean score calculation, and if more than 50% of the raters scored a response as incomprehensible, the mean score of that response was set to *not applicable* and excluded from further analysis. In cases where fewer than half of the raters marked a response as invalid, we retained the response and calculated the mean score based on the valid ratings. This approach followed our pre-registered rule and was chosen to avoid discarding potentially meaningful data based on isolated judgements. Disagreements among raters typically reflected differences in knowledge rather than inattentiveness. For example, one rater might not recognize that a “guiro” is a musical instrument and therefore mark the response as incomprehensible, while others rate it as valid.

GPT-4 ratings were initially rated on a 10–50 scale to allow for finer differentiation of DT creativity ratings, as specified in the prompt above. These scores, as well as the CLAUS scores, were subsequently rescaled to a 1–5 range to ensure comparability with human ratings and the outputs of OCSAI.

For explorations of potential language effects and to compare models with different language capabilities under standardized output conditions, all responses were translated into English using DeepL (https://www.deepl.com, accessed on 12 September 2024). The accuracy of the translation was examined with a systematic cross-check of 1000 randomly drawn responses across datasets. A student assistant majoring in Psychology and English Studies reviewed the translations and categorized them into three categories: (1) “I would translate it exactly the same”, (2) “Translation is inappropriate”, or (3) “Not sure”. This process allowed us to quantify translation accuracy and identify potential issues. In only 6.0% of these responses, a different translation approach was suggested, while in 2.4% of the cases, the translation appeared inappropriate (mostly in cases of spelling errors, ambiguous phrasing, or incomplete responses), indicating overall good translation quality. Nevertheless, DeepL has consistently demonstrated high translation quality in dependent evaluations, particularly for preserving context and idiomatic meaning (e.g., Kamaluddin et al. 2024; Linlin 2024). In our analysis, the original DeepL translations were used without further corrections in order to reflect the aims of an automated scoring pipeline.

### 2.6. Analysis Strategy

To investigate the relationship between human creativity ratings and LLM scores, we employed an integrative data analysis (IDA; Curran and Hussong 2009), which has multiple advantages compared to synthesizing summary statistics obtained from each dataset (e.g., ecological fallacies are prevented; Kaufmann et al. 2016). IDA is considered the gold standard for meta-analysis when datasets of individual participants are available (Rogozińska et al. 2017; Thomas et al. 2014) and has been suggested as an approach to integratively analyze data from multi-lab replications (van Aert 2022).

We used a one-step estimation approach (Curran and Hussong 2009; van Aert 2022) based on linear mixed models with random intercepts and random slopes. Specifically, the following model was estimated (cf. van Aert 2022):

(1) $$y_{ij}=\theta_{0i}+\lambda_{0j}+\theta_{1i}x_{ij}+\lambda_{1j}x_{ij}+{ℇ}_{ij},$$

with average human rating $y_{ij}$ for response j in dataset i, dataset-specific intercept $\theta_{0i}$, item-specific intercept $\lambda_{0j}$, dataset-specific linear slope $\theta_{1i}$, item-specific linear slope $\lambda_{1j}$, automated score $x_{ij}$ for response j in dataset i, and sampling error ${ℇ}_{ij}$ for response j in dataset i.

To account for variability across both studies and items, the dataset-specific intercepts and slopes ($\theta_{0i}$, $\theta_{1i}$) and the item-specific intercepts and slopes ($\lambda_{0j}$, $\lambda_{1j}$) were modeled as crossed random effects. We further assume a bivariate normal distribution N($\mu_{\theta }$, $\Sigma_{\theta }$) and a bivariate normal distribution ($\mu_{\lambda }$, $\Sigma_{\lambda }$) for the random effects across studies and items, respectively. The mean vectors μ incorporated the average intercepts and slopes, and the covariance matrices Σ included freely estimated variance and covariance parameters. Covariances between random effects across studies and random effects across items were fixed to zero. All models were estimated by means of restricted maximum likelihood (Bates et al. 2015).

The dependent variable $y_{ij}$ and the independent variable $x_{ij}$ represent the average human rating and the automated score, respectively, divided by their item-specific standard deviation within each dataset. These transformations ensured that the dataset-specific linear slopes $\theta_{1i}$ and the item-specific slopes $\lambda_{1j}$ could be interpreted analogously to the product-moment correlation coefficient (i.e., a measure for the linear relationship between variables with unit standard deviation). For that reason, we refer to the estimated coefficients ${\hat{\theta }}_{1i}$ and ${\hat{\lambda }}_{1j}$ simply as *correlations.* The employed model accounted for variability across both studies and items, addressing the heterogeneity inherent in the overall dataset. To quantify the heterogeneity in our results, prediction intervals were estimated, which indicate the range of expected correlation coefficients for future studies. To illustrate, a standard deviation of correlations across studies of 0.30 implies that 95% of the population correlations should vary between ± 1.96 × 0.30 = 0.59 units of the typical correlation for each of the studies. Detailed linear mixed model estimates are provided as Supplemental Analyses (see Tables S1–S3).

While linear mixed models were the preferred approach for analyzing associations between human ratings and LLM scores, a different approach was used when examining relationships among LLMs. Since all LLM scores were derived from the same dataset and thus shared identical study- and item-level variability, modeling random effects seemed unnecessary. Instead, Pearson correlation coefficients were computed to assess the agreement between LLMs, as they provide a more direct and interpretable measure of their linear association.

Analyses were performed using R (version 4.4.0, R Core Team) with the lme4 package (Bates et al. 2015) and the lmerTest package (Kuznetsova et al. 2017) for linear mixed models.

## 3. Results

### 3.1. Distribution of Creativity Ratings Across Studies

**Figure 1:**
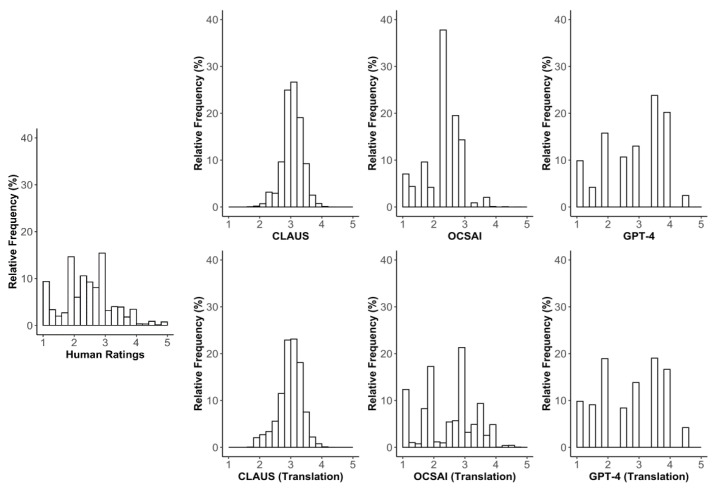
Distribution of human and large language model creativity ratings across studies.

Figure 1 provides an overview of the distributions of creativity ratings obtained from human raters and LLMs across studies. Distributions of LLM ratings are shown alternately for the original German dataset and the translated input.

Human ratings were concentrated in the lower to mid-range, forming a distribution that tends to be right-skewed. CLAUS scores exhibited a narrower and more symmetric distribution in both datasets, resembling a compressed normal distribution. OCSAI scores exhibited a distribution with a noticeable right skew in the German dataset, with specific values being clearly overrepresented, e.g., about 61.5% of scores fell into the *2.x* range in the German dataset (37.8% *2.3*), and the scores *1*, *2*, and *3* together accounted for 94.2% in the English dataset. GPT-4 scores exhibited a relatively wide distribution in both datasets, with certain scores being overrepresented, reflecting that, even when asking for a differentiated rating (originally 10–50), GPT-4 mostly still resorted to a less differentiated rating (i.e., *10*, *15*, *20*, *30*, *35*, *50*, which was then transformed to 1–5). Full descriptive statistics for each method are presented in the Supplemental Analyses (see Table S4).

### 3.2. Correlations Between Human and Large Language Model-Based Creativity Ratings

#### 3.2.1. Correlations Across All Responses

Correlations across the full dataset (individual response level, *N* = 48,507) between human and LLM creativity scores, derived from linear mixed models, are presented in Table 2, column 1.

For the German dataset, correlations with human ratings ranged from 0.46 (CLAUS) to 0.66 (OCSAI), while GPT-4 achieved a correlation of 0.55. Similar trends were observed for the translated dataset, with correlations ranging from 0.47 (CLAUS) to 0.60 (OCSAI) and GPT-4 exhibiting a slightly lower correlation of 0.54. Regarding prediction intervals, correlations for the German dataset ranged from 0.36 to 0.55 for CLAUS, from 0.48 to 0.84 for OCSAI, and from 0.32 to 0.79 for GPT-4. In the translated dataset, the intervals were generally wider, ranging from 0.24 to 0.71 for CLAUS, from 0.29 to 0.90 for OCSAI, and from 0.34 to 0.74 for GPT-4 (see Table 3).

Inter-model correlations revealed substantial agreement between LLMs. The strongest relationships were found between GPT-4 and its translated counterpart (*r* = 0.81), and between OCSAI and its translation (*r* = 0.72). Correlations between CLAUS and OCSAI ranged from *r* = 0.55 (translated dataset) to *r* = 0.57 (German dataset), reflecting moderate alignment. In contrast, GPT-4 displayed weaker associations with CLAUS (*r* = 0.48 and *r* = 0.56), but high correlations with OCSAI (*r* = 0.73 and *r* = 0.65) across the German and translated datasets, respectively.

#### 3.2.2. Correlations per Study

**Figure 2:**
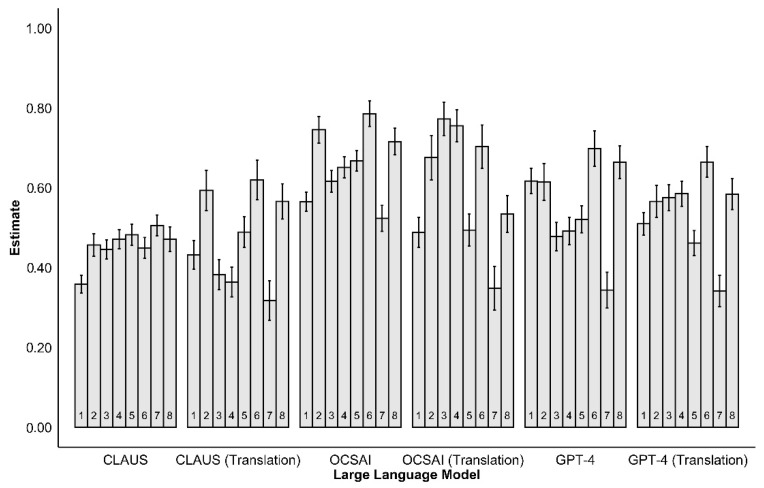
Correlations of creativity ratings by human and LLMs examined with linear mixed models per study. Notes. LLM = large language model. Error bars represent standard error of estimates.

Correlations between human ratings and LLM creativity ratings were examined separately for each study to explore their range across different study designs [see Figure 2 and Appendix A (Table A1)].

For CLAUS, correlations ranged from 0.36 (Study 1) to 0.51 (Study 7) for the German dataset and from 0.32 (Study 7) to 0.67 (Study 8) for the translated input, highlighting variability in its performance. For OCSAI, correlations with human ratings were consistently higher, ranging from 0.52 (Study 7) to 0.79 (Study 6) for the German dataset, and from 0.35 (Study 7) to 0.77 (Study 3) for the translations. GPT-4 exhibited moderate correlations on average but showed noticeable fluctuations across studies. Estimates ranged from 0.34 (Study 7) to 0.70 (Study 6) for the German dataset and from 0.34 (Study 7) to 0.66 (Study 6) for the translations.

#### 3.2.3. Correlations per Item

**Figure 3:**
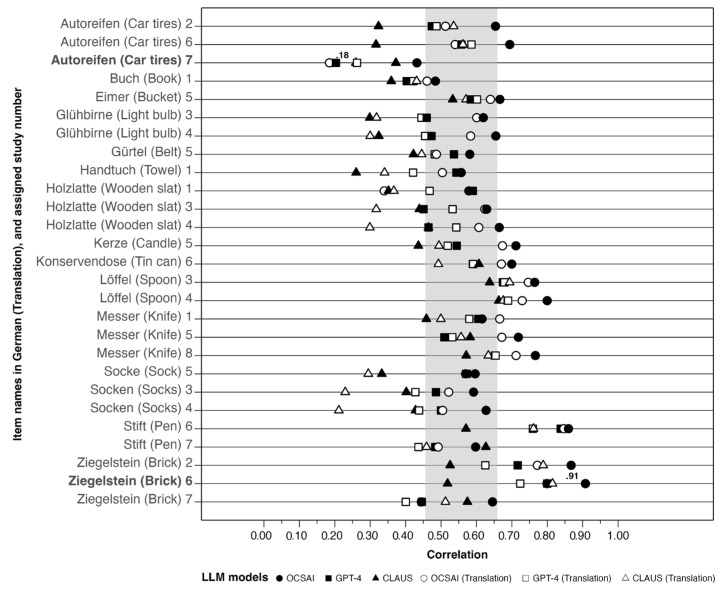
Correlations of human and LLM creativity ratings using LLMs per item. Notes. LLM = large language model. Shaded area reflects overall correlations across studies and LLMs from lowest (0.46, CLAUS) to highest (0.66, OCSAI). Items in bold represent lowest and highest single item/model correlation. German items were ordered alphabetically.

For a deeper insight into item-specific associations, we analyzed the correlations between human and LLM-based creativity ratings for each of the 15 items (see Figure 3). Some of the items were assessed in two or three studies. Across all models and studies, correlations of these 27 item-study combinations ranged from *r* = 0.18 [*Car tires*, Study 7, OCSAI (Translation)] to *r* = 0.91 (*Brick*, Study 6, OCSAI). Full descriptive statistics for each item are presented in the Supplemental Analyses (see Table S5).

For CLAUS, correlations in the German dataset exhibited moderate variability, spanning from *r* = 0.26 (*Towel*, Study 1) to *r* = 0.66 (*Spoon*, Study 4). Similarly, in the translated dataset, correlations ranged from *r* = 0.21 (*Socks*, Study 4) to *r* = 0.81 (*Brick*, Study 6). Notably, only 14.8% of the items in the German dataset achieved correlations of *r* ≥ 0.60, compared to 22.2% in the translated dataset. For OCSAI, correlations in the German dataset ranged from *r* = 0.43 (*Car tires*, Study 7) to *r* = 0.91 (*Brick*, Study 6), with 51.9% of the items achieving correlations of *r* ≥ 0.65. In the translated dataset, correlations ranged from *r* = 0.18 (*Car tires*, Study 7) to *r* = 0.85 (*Pen*, Study 6), with 37.0% of items reaching correlations of *r* ≥ 0.65, reflecting slightly lower consistency compared to the German datasets. For GPT-4, correlations in the German dataset ranged from *r* = 0.20 (*Car tires*, Study 7) to *r* = 0.84 (*Pen*, Study 6). Of these, 22.2% achieved correlations of *r* ≥ 0.65, while 64.2% reached *r* ≥ 0.50. In the translated dataset, correlations ranged from *r* = 0.26 (*Car tires*, Study 7) to *r* = 0.76 (*Pen*, Study 6). Here, 18.5% of items achieved correlations of *r* ≥ 0.65, while 58.0% reached *r* ≥ 0.50, showing a slight decline compared to the German dataset.

### 3.3. Exploring the Effectiveness of Large Language Model Scorings by Means of Rater Statistics

To further gauge the suitability of LLMs as raters in the context of creativity research, we computed rater statistics at the response-level (equivalent to item statistics) for those five datasets that have three or more raters (Studies 1, 5, 6, 7, 8). We computed rater-total correlations as an index of the discriminatory power of LLM methods compared to the human ratings. Table 4 summarizes the relative frequencies of cases where LLMs were identified as the weakest or best rater, or neither in terms of their rater-total correlation. Detailed results are available on the OSF (Table S6).

LLMs were identified as the weakest rater in 50.0% of cases, fell into the neither/nor category (neither weakest nor best) in 43.3%, and were considered the best rater in 6.7% with a weighted overall mean rater-total statistic of 0.71. CLAUS was identified as the weakest in 80.0% of cases and fell into the neither/nor category in 20.0%, with no instances of being classified as the best. Its mean rater-total correlation was 0.66 for the original German input and 0.69 for the translated input. OCSAI was the weakest in 20.0% of cases for the original German input and in 40.0% for the translated input. It was the best rater in 40.0% of cases for the original input, with no such cases for the translated input. Its mean rater-total correlation was 0.80 for the original input and 0.71 for the translated input. GPT-4 was the weakest in 20.0% of cases for the original input and 60.0% for the translated input, falling into the neither/nor category in the remaining cases (80.0% for the original German dataset and 40.0% for the translated input). Its mean rater-total correlation was 0.74 for the original German and 0.72 for the translated input. Mean rater-total correlation without LLMs was 0.74.

## 4. Discussion

Recent advances in machine learning are more and more applied to evaluate creativity in human responses and allow for an automated and less laborious scoring of such tasks (Goecke et al. 2024; Wahbeh et al. 2024; Yang et al. 2024; Zielińska et al. 2023). However, systematic comparisons of these scoring approaches remain scarce, particularly in languages other than English. Even fewer studies provide insights into the robustness of automated scoring across different studies and items. This work addresses these gaps by presenting a comprehensive analysis of three current LLM-based creativity scoring methods (i.e., CLAUS, OCSAI, GPT-4) applied to a large dataset of German AUT responses compiled across eight datasets from five research groups. Across the entire dataset of about 50,000 responses, we find that LLM-based models predict human creativity scores with substantial correlations in the range of 0.46 to 0.66. These findings speak directly to our first research question, which focused on the extent to which LLM-based scoring aligns with human creativity judgements.

Our findings add to the evidence that LLM-based predictions are clearly better than those previously obtained with semantic distance measures (Organisciak et al. 2023; Patterson et al. 2023). They are consistent with previous results that observed correlations for OCSAI between 0.42 and 0.72 depending on the item (Zielińska et al. 2023). Higher response-level correlations around 0.80 and higher have only been reported previously when testing the prediction of held-out data within the same English dataset (Luchini et al. 2025a; Organisciak et al. 2023; Patterson et al. 2023), whereas this study tested the autoscoring performance of LLMs in non-English data from independent datasets. In general, these findings suggest that LLM-based scoring can be conducted with German DT data. However, there are a number of nuances that need to be carefully considered when deciding on automated scoring with a specific LLM. In the following, we discuss to what extent findings depend on the LLM, translation of responses, and how findings generalize across studies and items.

### 4.1. Comparison of Large Language Models

We compared the creativity rating performance of CLAUS, OCSAI, and GPT-4 across our fairly large dataset against human ratings. This is specifically interesting from a conceptual multi-trait-multi-method (MTMM) perspective (cf. Campbell and Fiske 1959). From this perspective, we treat originality as a focal *trait* and distinguish among different *methods* of scoring responses—namely, human raters versus algorithmic scoring through distinct LLMs. In line with this framework, high agreement (i.e., correlations) across methodologically distinct methods (e.g., human and LLM-based) when assessing the same trait (=originality) would support convergent validity, while systematic differences between methods would indicate divergence. As stated by Campbell and Fiske (1959, p. 83), reliability and “validity can be seen as regions of a continuum”, and in this sense, “validity is represented in the agreement between two attempts to measure the same trait through maximally different methods”.

At this point, it is important to clarify the structure of our measurement design. We consider rater types (human vs. LLMs) as competing instantiations of one method dimension. In our study design, the single AUT items might be understood as a second method dimension. Validity evidence hinges on the extent to which trait-relevant variance (originality differences) is consistent across both item- and rater-based methodological variations.

We found that all three models predicted human creativity ratings at substantial levels, however, with serious heterogeneity. OCSAI was the top-performing model in most instances. Its performance remained satisfying across the eight included studies and nearly all 15 distinct items. In contrast, CLAUS and GPT-4 showed substantial but relatively smaller correlations with the human ratings, along with greater heterogeneity across items. This means that OCSAI aligns most closely with human creativity ratings, offering stronger evidence for convergent validity, while CLAUS and GPT-4 show weaker and more variable agreement—highlighting method effects across items and models. This finding addressed our second research question, which aimed at determining which LLM method performs best when predicting human creativity judgements.

From a judge response theory perspective (Myszkowski and Storme 2019), individual raters—whether humans or LLMs—can be conceptualized as indicators of an underlying latent construct (e.g., creative performance or originality in our case). This framework supports our interpretation that convergent validity is indicated when different methods yield similar scores across items. Nonetheless, the degree of independence among methods (as emphasized by Campbell and Fiske 1959) remains a critical point of discussion. While our scoring methods are not completely independent (since both humans and LLMs rely on the same participant responses), they do represent distinct procedures, which we argue justifies a cautious interpretation of our results as evidence for convergent validity.

The advantage of OCSAI likely stems from the fact that it combines the power and versatility of GPT-4o mini with additional fine-tuning (Organisciak et al. 2023). Hence, the increased performance beyond that of the GPT-4 base model was to be expected. Nevertheless, the performance of the GPT-4 base model was still remarkable given that it did not rely on any fine-tuning, but instead just a simple and straightforward prompt asking the model to evaluate the creativity of responses based on a short definition. This highlights the base model’s already substantial capacity to approximate human-like creativity assessments, and, given the rapid development of LLMs, it is to be expected that this capability might increase further in the near future.

The results suggested that GPT-4 tended to outperform CLAUS, especially when rating the original German responses; for the translated responses, CLAUS showed higher correlations with the human ratings than GPT-4 in four out of eight studies. This pattern underscores the potential influence of linguistic and methodological nuances in automated scoring approaches (Yang et al. 2024). This is further emphasized through the result that CLAUS performed very well for a number of items (and hence different prompts), but its performance dropped considerably for others. In contrast to that, GPT-4 showed a more consistent performance across items (*r* ≥ 0.40, except for one item). This variability is further reflected in the prediction intervals, which indicate considerable uncertainty in the expected range of correlations between human and LLM creativity ratings across future studies, with interval widths ranging from 0.10 to 0.31 depending on the model and language condition. Such variability within and across models is central to our third research question, which examined generalizability across datasets and items.

Viewed through the MTMM perspective, these results illustrate why employing multiple methods to assess creativity might remain crucial in the near future. OCSAI’s consistently higher correlations with human ratings suggest that it captures the target trait the best, but from our view, it should be understood as merely one method to approximate what is to be measured in the first place. After all, human ratings are also made up of several “methods”: different raters. Although CLAUS and GPT-4 showed somewhat lower consistency in their performance, the correlations were still substantial and also higher than previous automated scoring approaches using semantic distances (Forthmann et al. 2017b; Forthmann and Doebler 2022). Future research can build on these findings by examining more granular aspects of how different LLMs or fine-tuning strategies influence the ratings of creativity responses.

### 4.2. Effects of Response Translation

The translation of responses to English had only a limited effect on the correlations between LLM and human creativity ratings. CLAUS tended to benefit from the translation as it slightly increased correlations in five out of eight studies, whereas for OCSAI and GPT-4, translations reduced correlations in the same amount of studies. Taking a closer look at the LLMs themselves and their correlation with their translated counterparts, we observed varying degrees of consistency. GPT-4 exhibited the highest correlation between the original and translated versions, followed by OCSAI and CLAUS. These findings suggest that while GPT-4 demonstrates relatively stable performance across languages, translation may still introduce some variability. Compared to previous studies that reported near-complete interchangeability of OCSAI scores across languages (e.g., Zielińska et al. 2023), our results indicate that multilingual performance depends on the specific model architecture. Notably, CLAUS, despite being developed for creative scoring across multiple languages, showed the lowest cross-linguistic correlation. This suggests that its performance may be more sensitive to the input language than previously assumed (Luchini et al. 2025a; Patterson et al. 2023). These findings highlight that while LLMs offer strong multilingual capabilities, translation effects should still be considered, particularly for models with less explicit multilingual training, and for languages that are currently less represented in cross-language training and less accessible for model fine-tuning.

### 4.3. Variation Across Items and Studies

A closer inspection of the relationships at the level of single studies or items corroborated the general robustness of our findings but also pointed to notable variability. For example, while some AUT items like *Spoon* yielded high correlations with human ratings for all LLMs (*r* > 0.60), other items like *Book* had low correlations for all models (*r* < 0.50), and yet other items like *Socks* implied a large range in correlations across models (*r* = 0.21 to *r* = 0.63). The reasons for this variability were not fully explored. Differences across models could be due to specific characteristics of the item, responses, or study. For example, AUT items are known to differ in their semantic richness, and those with more sparse semantic networks (e.g., *Pen*) elicit more creative responses (Beaty et al. 2022), which may increase variance and thereby enhance correlations. Higher correlations could also be due to a high rate of uncreative responses (e.g., in studies applying “be-fluent” instructions), which typically yield excellent agreement across raters but also LLMs. Finally, item-related variability across models may partly be due to how these concepts are represented in the LLMs or the extent of exposure to them during fine-tuning. For example, CLAUS showed the highest variance in correlations across items, ranging between *r* = 0.21 and *r* = 0.81. Study characteristics that might also explain some of the found variability in the items include varying characteristics of the samples themselves (adolescents versus adults), the design choices (e.g., un-proctored versus proctored) and slightly different rating instructions [e.g., creativity rating emphasizing novelty and effectiveness of ideas (Diedrich et al. 2015)]. Since LLM-based creativity scores were always generated in the context of the respective item, including the item as a random effect in our models serves to adjust for overall item-level differences in scoring, without removing meaningful item-response interactions. The considerable variation in item-level performance across models—particularly the observation that some items yield substantially lower correlations—raises important questions about the generalizability of current scoring approaches. A more systematic investigation of such variability, including whether it is driven by semantic features of items, instruction types, or sample characteristics, represents a valuable direction for future research.

### 4.4. Can Large Language Models Replace Human Raters?

Our findings demonstrate that LLMs are able to predict human creativity ratings to a substantial degree, but also that considerable shares of variance are unaccounted for. Most of our analyses used the average human creativity ratings as the criterion, but human raters also did not agree perfectly—which is also not to be expected. Therefore, we additionally computed rater statistics at the response-level to explore how LLM-ratings compare to individual human raters. These analyses revealed that the discriminatory power of LLMs is very similar to that of human raters, referring to how well a rater or model can distinguish between more versus less creative responses (cf. Silvia 2008). In fact, in 80.0% of cases, OCSAI was equally or even more discerning than the human raters. Similarly, in 80.0%, ratings by GPT-4 were also comparable to those of the human raters. Ratings by CLAUS showed similar discriminatory power as the human raters in 20.0%. These rater statistics transfer the logic of item statistics—such as item-total correlations from classical test theory—to the level of raters, treating each rater (or model) as an “item” whose consistency with the overall judgement can be quantified. While exploratory in nature, this analysis suggested that CLAUS exhibited notably weaker discriminatory ability in this framework, pointing to potential limitations in its capacity to align with human judgements. As a potential qualification of this analysis, the relevant total was determined by a larger number of human raters than LLMs; on the other hand, LLMs evaluated creativity on a more differentiated scale than human raters who only used a five-point scale. Taken together, our findings suggest that ratings by LLMs—especially OCSAI and GPT-4—can approximate the consistency of human raters. This does not necessarily mean that LLM ratings can directly replace human ratings, but at least replace single human raters, as we need to assume that a single LLM is about as unreliable as a single human rater (Ceh et al. 2022). Still, from now on, we may consider teaming up different LLMs or LLM agents with different personas (Huang et al. 2024; Vinchon et al. 2023) to achieve high overall reliability. This reinforces the idea that using multiple raters in tandem with LLMs could strengthen the overall creativity assessment. Importantly, we observed that LLMs have no big issues with orthographically incorrect or even partly incomplete responses, which used to be an important benefit of human raters (e.g., compared to earlier approaches based on semantic distance). Hence, just like human raters, LLMs in this study rated most responses but also usually flagged invalid responses such as those that were entirely incomprehensible.

### 4.5. Limitations and Future Directions

A central goal of this study was to explore the feasibility of automatic methods to increase both efficiency and objectivity in creativity scoring. In practice, we ran into some issues during LLM-based scoring that need further attention to ensure a fully automatic, objective scoring process. CLAUS and OCSAI both come with graphical user interfaces that support convenient upload of response data; however, both methods could not handle data as large as ours (initially over 60,000 responses). OCSAI can also be accessed via an API which deals better with larger data. Curiously, OCSAI returned consistently lower ratings for some items (i.e., *Brick* and *Knife*), maybe related to its fine-tuning process, which can be an issue when correlating across responses from different items, but this was accounted for in our analysis. For GPT-4, the way the model is prompted still introduces a degree of subjectivity. Just as an inadequate prompt may lower an LLM’s automated scoring performance, we expect potential for further increasing its performance using soft prompting optimization or engineering of hard prompts. Hence, systematic analyses of the effect of different prompting methods on automated scoring methods represent an important avenue for future research. Moreover, we observed that scorings are not necessarily stable across time and conditions such as how many responses are scored at once. Therefore, we ended up scoring responses individually via the API (i.e., no batches for the GPT-4 approach, individual prompt per line), which was a costly procedure both with respect to time and money. Notably, commercial LLMs are always subject to change without notice, thereby undermining the reproducibility of scoring. For the future, it would be interesting to also consider other powerful LLMs such as Claude, Gemini, as well as open-source LLMs like LLaMA (Grattafiori et al. 2024; Touvron et al. 2023). When using base model LLMs, verbatim prompts should be disclosed together with model parameters (e.g., temperature) just like it is recommended for human rating instructions.

As a limitation of this study, we focused on a single DT task, the popular AUT (Saretzki et al. 2024b). At the same time, this has been the biggest effort in leveraging German AUT data to date. An increasing number of studies suggests that LLMs perform well also for several other DT tasks such as creative story writing, metaphor production, real-world problem solving, scientific ideation, and even for DT in the visual domain (Cropley and Marrone 2022; DiStefano et al. 2024; Luchini et al. 2025a; Organisciak et al. 2023; Patterson et al. 2024b). So far, most of this evidence relies on LLMs that were fine-tuned to a specific task. Our findings suggest that powerful base models like GPT-4 already outperform traditional semantic distance approaches in assessing creativity. Given the rapid pace of development in LLMs, this gap may narrow quickly, as more advanced models are expected to improve their ability to assess creative performance across multiple tasks and languages without requiring fine-tuning. Hence, future work should examine how automatic scoring performance varies across different types of DT tasks, as using different DT tasks likely supports construct coverage. As another limitation, our analyses only focused on what method maximized the relationship with human raters. This approach does not consider how scoring methods affect validity evidence such as in terms of predicting creative behavior and achievement (Saretzki et al. 2024b), and more reliable measures are not necessarily more valid (Benedek et al. 2013). One can further envision hybrid scoring approaches, where humans remain in the loop to ensure quality standards. Moreover, by reducing the costly human resources in the rating process, LLMs could be tasked to engage in more complex rating procedures such as assessing different quality dimensions (e.g., novelty, effectiveness, remoteness) which then could be combined to more powerful compound scores or studied separately.

## 5. Conclusions

Scoring DT responses with LLMs is nearly as reliable as human ratings and thus a promising alternative to subjective creativity scoring, with a growing number of open-access resources for researchers to facilitate automated creativity assessment for a range of DT tasks. Our findings demonstrate that specific LLMs work increasingly well for German AUT data usually without the need to translate responses to English prior to scoring. Once reproducible scoring pipelines are established, LLM-based creativity scoring can be a powerful tool to complement and replace human scoring and thereby expedite and standardize creativity research.

## Figures and Tables

**Table 1:** Key characteristics of included studies.

ID	Reference	*N*	Items	Responses	Raters	ICC [95% CI]
1	Weiss et al. 2024	328	4	11,599	3	0.786 [0.779, 0.793]
2	Knopf and Lechner 2025	443	2	2445	2	0.810 [0.795, 0.825]
3	Jaggy et al. 2025	129	4	2835	2 ^1^	0.804 [0.744, 0.857]
4	Jaggy et al. 2025	218	4	5042	2 ^1^	0.814 [0.785, 0.909]
5	Saretzki et al. 2024b	300	5	7691	6	0.855 [0.850, 0.860]
6	Lebuda and Benedek 2024	425	4	13,244	6	0.813 [0.808, 0.818]
7	Benedek and Lebuda 2024	317	3	5675	6	0.808 [0.800, 0.815]
8	Forthmann et al. 2017a	160	1	860	3	0.902 [0.890, 0.913]
Total	-	2320	27 ^2^	49,391	30	-

Notes. The number of participants and responses reflect data prior to the exclusion of 884 invalid responses and 30 participants without valid responses during data processing. ICC values represent item-specific ICC(3,k), except for Studies 3 and 4, which report ICC(1,k) due to the two-rater combination setup. ^1^ Five raters in different two-rater-combinations (every response has been rated by two raters). ^2^ Number of distinct items: 15. Note that “Sock” and “Socks” were treated as separate items.

**Table 2:** Overall correlation matrix of human and LLM creativity ratings.

	1. ^1^	2.	3.	4.	5.	6.
1. Human Ratings	-					
2. CLAUS	0.46	-				
3. CLAUS (Translation)	0.47	0.54	-			
4. OCSAI	0.66	0.57	0.63	-		
5. OCSAI (Translation)	0.60	0.45	0.55	0.72	-	
6. GPT-4	0.55	0.48	0.54	0.73	0.59	-
7. GPT-4 (Translation)	0.54	0.45	0.56	0.69	0.65	0.81

Notes. LLM = large language model. All correlations reported were significant at *p* < .001. ^1^ lme4 Syntax for estimating the model in Equation (1) (i.e., the correlation between human and LLM creativity ratings): HumanRatings ~ LLM + (1 + LLM | Study) + (1 + LLM | Item).

**Table 3:** Prediction intervals for human-LLM creativity rating correlations.

LLM	Estimate	*SE*	Prediction Interval		Interval Range
			LL	UL	
CLAUS	0.46	0.04	0.36	0.55	0.10
CLAUS (Translation)	0.47	0.06	0.24	0.71	0.23
OCSAI	0.66	0.04	0.48	0.84	0.18
OCSAI (Translation)	0.60	0.07	0.29	0.90	0.31
GPT-4	0.55	0.05	0.32	0.79	0.24
GPT-4 (Translation)	0.54	0.05	0.34	0.74	0.20

Notes. LLM = large language model.

**Table 4:** Rater statistics including LLMs.

	LLM(s) as Weakest Rater	LLM(s) Neither Weakest nor Best	LLM(s) as Best Rater	Mean LLM-Total Correlation
CLAUS	80.0%	20.0%	-	0.66
CLAUS (Translation)	80.0%	20.0%	-	0.69
OCSAI	20.0%	40.0%	40.0%	0.80
OCSAI (Translation)	40.0%	60.0%	-	0.71
GPT-4	20.0%	80.0%	-	0.74
GPT-4 (Translation)	60.0%	40.0%	-	0.72
Average	50.0%	43.3%	6.7%	0.71

Notes. LLMs = large language models. Relative frequencies always refer to the relevant total of the number of rater statistics including datasets with three or more raters (i.e., Studies 1, 5, 6, 7, and 8).

## Data Availability

All files for analyses are available on the Open Science Framework: https://osf.io/eaqtb, accessed on 5 September 2024.

## Notes

1 Three additional datasets were initially considered in the pre-registration but not included in the analyses. Weiss et al. (2023) was excluded because its ratings focused solely on assessing the appropriateness of responses. Kleinkorres et al. (2021) and Forthmann and Doebler (2022) were excluded because they were part of the German training dataset for the fine-tuned LLMs (see Patterson et al. 2023), which undermines the independence of the test data.

2 We used the GPT-4 model instead of the pre-registered GPT-4o, as GPT-4o was not available for the data volume at the time of data collection.

3 Note: GPT is available both through OpenAI’s chat interface and via a paid Application Programming Interface (API). While the chat interface allows for manual interactions, the API is recommended for processing larger datasets. The R code provided in the OSF Supplemental Material documents the procedure used in this study.

## Supplementary Materials

The following supporting information can be downloaded at: https://www.mdpi.com/article/10.3390/jintelligence13060064/s1.

## Appendix A

**Table A1:** Study level correlations of human and LLM creativity ratings using linear mixed models.

Study	Responses	CLAUS		OCSAI		GPT-4	
		German	Translation	German	Translation	German	Translation
Across Studies	48,507	0.46	0.47	0.66	0.60	0.55	0.54
1	11,151	0.36	0.43	0.57	0.49	0.62	0.51
2	2319	0.46	0.59	0.75	0.68	0.62	0.57
3	2733	0.45	0.38	0.62	0.77	0.48	0.58
4	4917	0.47	0.36	0.65	0.76	0.49	0.59
5	7608	0.48	0.49	0.67	0.49	0.52	0.46
6	13,244	0.45	0.62	0.79	0.70	0.70	0.66
7	5675	0.51	0.32	0.52	0.35	0.34	0.34
8	860	0.47	0.67	0.72	0.53	0.66	0.58

Notes. LLM = large language model. All correlations reported were significant at *p* < .001.

**Table A2:** Item level correlations of human and LLM creativity ratings using linear mixed models.

ID	Item		CLAUS		OCSAI		GPT-4	
		*N*	German	Translation	German	Translation	German	Translation
1	Buch (Book)	1825	0.36	0.43	0.48	0.46	0.40	0.42
1	Handtuch (Towel)	4493	0.26	0.34	0.56	0.50	0.54	0.42
1	Holzlatte (Wooden slat)	1803	0.35	0.37	0.58	0.34	0.59	0.47
1	Messer (Knife)	3030	0.46	0.50	0.62	0.67	0.61	0.58
2	Autoreifen (Car tires)	1078	0.32	0.54	0.65	0.51	0.47	0.49
2	Ziegelstein (Brick)	1241	0.53	0.79	0.87	0.77	0.72	0.63
3	Glühbirne (Light bulb)	517	0.30	0.32	0.62	0.60	0.46	0.44
3	Holzlatte (Wooden slat)	921	0.44	0.32	0.63	0.62	0.45	0.53
3	Löffel (Spoon)	636	0.64	0.69	0.76	0.75	0.67	0.68
3	Socken (Socks)	659	0.40	0.23	0.59	0.52	0.49	0.43
4	Glühbirne (Light bulb)	1009	0.32	0.30	0.65	0.58	0.47	0.46
4	Holzlatte (Wooden slat)	1467	0.46	0.30	0.66	0.61	0.46	0.54
4	Löffel (Spoon)	1166	0.66	0.68	0.80	0.73	0.69	0.69
4	Socken (Socks)	1275	0.43	0.21	0.63	0.50	0.50	0.44
5	Eimer (Bucket)	1751	0.53	0.57	0.67	0.64	0.58	0.60
5	Gürtel (Belt)	1476	0.42	0.45	0.58	0.49	0.54	0.48
5	Kerze (Candle)	1294	0.44	0.49	0.71	0.67	0.54	0.52
5	Messer (Knife)	1316	0.58	0.56	0.72	0.67	0.51	0.53
5	Socke (Sock)	1771	0.33	0.29	0.60	0.57	0.57	0.57
6	Autoreifen (Car tires)	3182	0.32	0.56	0.69	0.54	0.56	0.59
6	Konservendose (Tin can)	3529	0.61	0.49	0.70	0.67	0.59	0.59
6	Stift (Pen)	3154	0.57	0.76	0.86	0.85	0.84	0.76
6	Ziegelstein (Brick)	3379	0.52	0.81	0.91	0.80	0.80	0.72
7	Autoreifen (Car tires)	1969	0.37	0.26	0.43	0.18	0.20	0.26
7	Stift (Pen)	1742	0.63	0.46	0.60	0.49	0.48	0.44
7	Ziegelstein (Brick)	1964	0.57	0.51	0.65	0.44	0.45	0.40
8	Messer (Knife)	860	0.57	0.63	0.77	0.71	0.65	0.66

Notes. All correlations reported were significant at *p* < .001.
