# Supplementary material for: Scoring German Alternate Uses Items Applying Large Language Models
Source: J Intell. 2025 May 29;13(6):64. doi: 10.3390/jintelligence13060064 (PMC12194149; doi:10.3390/jintelligence13060064)
Supplement: Supplementary file 1 [file jintelligence-13-00064-s001.zip › jintelligence-3492450-supplementary.pdf]

Supplemental Analyses

Scoring German Alternate Uses Items Applying Large Language Models

Table S1. CLAUS Linear mixed models.

a *Linear mixed model estimates (random and fixed effects) of CLAUS*

CLAUS – Random Effects				
Groups	Name	Variance	*SD*	Corr
Item	(Intercept)	1.546	1.24	
	CLAUS	0.013	0.11	-.87
Study	(Intercept)	0.373	0.61	
	CLAUS	0.002	0.05	-.46
Residual		0.812	0.90	

CLAUS – Fixed Effects				
	Estimate	*SE*	*df*	*t*
(Intercept)	-1.20	0.39	19.59	-3.03**
CLAUS	0.46	0.04	19.10	13.01***

Correlation of Fixed Effects: -.76

b *Linear mixed model estimates (random and fixed effects) of CLAUS (Translation)*

CLAUS (Translation) – Random Effects				
Groups	Name	Variance	*SD*	Corr
Item (Translation)	(Intercept)	1.315	1.15	
	CLAUS (Translation)	0.020	0.14	-.77
Study	(Intercept)	1.038	1.02	
	CLAUS (Translation)	0.014	0.12	-.68
Residual		0.745	0.86	

CLAUS (Translation) – Fixed Effects				
	Estimate	*SE*	*df*	*t*
(Intercept)	-0.60	0.47	14.38	-1.27
CLAUS (Translation)	0.47	0.06	15.12	8.31***

Correlation of Fixed Effects: -.72

Table S2. OSCAI Linear mixed models.

a *Linear mixed model estimates (random and fixed effects) of OCSAI*

OCSAI – Random Effects				
Groups	Name	Variance	*SD*	Corr
Item	(Intercept)	0.388	0.62	
	OCSAI	0.007	0.08	-.69
Study	(Intercept)	0.318	0.56	
	OCSAI	0.009	0.09	-.42
Residual		0.568	0.75	

OCSAI – Fixed Effects				
	Estimate	*SE*	*df*	*t*
(Intercept)	0.60	0.26	14.74	2.33*
OCSAI	0.66	0.04	12.10	16.67***

Correlation of Fixed Effects: -.52

b *Linear mixed model estimates (random and fixed effects) of OCSAI (Translation)*

OCSAI (Translation) – Random Effects				
Groups	Name	Variance	*SD*	Corr
Item (Translation)	(Intercept)	0.499	0.71	
	OCSAI (Translation)	0.020	0.14	-.46
Study	(Intercept)	0.978	0.99	
	OCSAI (Translation)	0.025	0.16	-.74
Residual		0.659	0.81	

OCSAI (Translation) – Fixed Effects				
	Estimate	*SE*	*df*	*t*
(Intercept)	1.08	0.40	10.34	2.73*
OCSAI (Translation)	0.60	0.07	11.91	8.89***

Correlation of Fixed Effects: -.67

Table S3. GPT-4 Linear mixed models.

a *Linear mixed model estimates (random and fixed effects) of GPT-4*

GPT-4 – Random Effects				
Groups	Name	Variance	*SD*	Corr
Item	(Intercept)	0.237	0.49	
	GPT-4	0.012	0.11	-.78
Study	(Intercept)	0.476	0.69	
	GPT-4	0.015	0.12	-.57
Residual		0.681	0.83	

GPT-4 – Fixed Effects				
	Estimate	*SE*	*df*	*t*
(Intercept)	1.55	0.28	10.30	5.62***
GPT-4	0.55	0.05	12.70	10.58***

Correlation of Fixed Effects: -.62

b *Linear mixed model estimates (random and fixed effects) of GPT-4 (Translation)*

GPT-4 (Translation) – Random Effects				
Groups	Name	Variance	*SD*	Corr
Item (Translation)	(Intercept)	0.247	0.50	
	GPT-4 (Translation)	0.010	0.10	-.31
Study	(Intercept)	0.521	0.72	
	GPT-4 (Translation)	0.011	0.10	-.55
Residual		0.722	0.85	

GPT-4 (Translation) – Fixed Effects				
	Estimate	*SE*	*df*	*t*
(Intercept)	1.78	0.29	10.33	6.18***
GPT (Translation)	0.54	0.05	12.91	11.79***

Correlation of Fixed Effects: -.49

Notes. P-Values for fixed effects were calculated using Satterthwaite's approximation.

Model equation: HumanRatings ~ LLM + (1 + LLM | Study) + (1 + LLM | Item).

Table S4. *Descriptive statistics per study across measures.*

	Min	Max	*M*	*SD*
**Human Ratings**	**1.00**	**5.00**	**2.45**	**0.77**
1	1.00	5.00	2.16	0.78
2	1.00	5.00	2.29	0.99
3	1.00	5.00	2.66	1.12
4	1.00	5.00	2.60	1.13
5	1.00	5.00	2.79	0.59
6	1.00	4.67	2.49	0.62
7	1.00	4.83	2.43	0.68
8	1.00	5.00	2.51	0.93
**CLAUS**	**1.63**	**4.29**	**3.06**	**0.31**
1	1.78	4.15	3.00	0.29
2	1.74	3.82	2.97	0.29
3	1.78	4.01	3.01	0.32
4	1.78	4.14	2.99	0.33
5	1.85	4.29	3.18	0.31
6	1.68	4.26	3.06	0.31
7	1.63	4.09	3.13	0.30
8	2.05	4.18	3.03	0.31
**CLAUS (Translation)**	**1.73**	**4.27**	**2.97**	**0.39**
1	1.76	4.06	2.98	0.31
2	1.79	4.08	2.83	0.45
3	1.76	4.01	3.00	0.37
4	1.77	4.10	2.98	0.38
5	1.81	4.27	3.08	0.32
6	1.73	4.26	2.93	0.43
7	1.77	4.18	3.00	0.40
8	2.17	4.02	3.04	0.31
**OCSAI**	**1.00**	**4.70**	**2.32**	**0.60**
1	1.00	4.30	2.26	0.50
2	1.00	3.70	2.08	0.65
3	1.00	4.30	2.17	0.63
4	1.00	4.00	2.16	0.64
5	1.00	4.70	2.53	0.53
6	1.00	4.30	2.31	0.62
7	1.00	4.70	2.43	0.61
8	1.00	4.00	2.35	0.54
**OCSAI (Translation)**	**1.00**	**5.00**	**2.54**	**0.90**
1	1.00	4.60	2.44	0.83
2	1.00	4.50	2.26	1.04
3	1.00	4.80	2.65	0.72
4	1.00	4.40	2.64	0.74
5	1.00	5.00	2.74	0.84
6	1.00	4.80	2.53	0.94
7	1.00	5.00	2.69	0.93
8	1.00	4.00	1.76	0.63

**GPT-4**	**1.00**	**5.00**	**2.90**	**0.99**
1	1.00	5.00	2.60	0.88
2	1.00	4.50	2.72	1.08
3	1.00	4.50	2.65	1.04
4	1.00	4.50	2.65	1.06
5	1.00	4.50	3.28	0.82
6	1.00	4.50	2.97	1.01
7	1.00	4.50	3.12	0.94
8	1.00	4.50	2.85	1.07
**GPT-4 (Translation)**	**1.00**	**4.50**	**2.77**	**1.03**
1	1.00	4.50	2.47	0.90
2	1.00	4.50	2.72	1.11
3	1.00	4.50	2.63	1.08
4	1.00	4.50	2.64	1.08
5	1.00	4.50	3.10	0.93
6	1.00	4.50	2.79	1.06
7	1.00	4.50	3.00	1.01
8	1.00	4.50	2.69	1.13

Notes. Weighted overall descriptive statistics are presented above each section and highlighted in bold.

Table S5. *Descriptive statistics per item across measures.*

ID	Item (Translation)	Rating	Min	Max	*M*	*SD*
1	Buch (Book)	Human Ratings	1.00	5.00	2.19	0.76
		CLAUS	1.80 (1.76)	3.97 (3.94)	2.99 (2.95)	0.30 (0.35
		OCSAI	1.00 (1.00)	3.70 (4.50)	2.33 (2.79)	0.48 (0.77)
		GPT-4	1.00 (1.00)	4.50 (4.50)	2.67 (2.48)	0.81 (0.81)
1	Handtuch (Towel)	Human Ratings	1.00	5.00	2.25	0.75
		CLAUS	1.78 (1.77)	3.93 (4.06)	3.04 (2.97)	0.28 (0.34)
		OCSAI	1.00 (1.00)	4.30 (4.60)	2.38 (2.87)	0.51 (0.63)
		GPT-4	1.00 (1.00)	5.00 (4.50)	2.87 (2.71)	0.84 (0.92)
1	Holzlatte (Wooden Slat)	Human Ratings	1.00	5.00	2.11	0.80
		CLAUS	2.06 (2.13)	3.74 (3.82)	3.05 (3.07)	0.25 (0.23)
		OCSAI	1.00 (1.00)	3.70 (3.70)	2.08 (2.41)	0.43 (0.60)
		GPT-4	1.00 (1.00)	4.00 (4.50)	2.16 (2.20)	0.70 (0.75)
1	Messer (Knife)	Human Ratings	1.00	5.00	2.03	0.80
		CLAUS	2.04 (2.14)	4.15 (4.05)	2.91 (2.96)	0.31 (0.28)
		OCSAI	1.00 (1.00)	3.70 (4.00)	2.14 (1.61)	0.50 (0.55)
		GPT-4	1.00 (1.00)	4.50 (4.50)	2.42 (2.27)	0.95 (0.93)
2	Autoreifen (Car Tires)	Human Ratings	1.00	5.00	2.73	0.82
		CLAUS	1.80 (1.79)	3.79 (3.83)	3.01 (3.02)	0.29 (0.36)
		OCSAI	1.00 (1.00)	3.70 (4.50)	2.41 (3.10)	0.54 (0.82)
		GPT-4	1.00 (1.00)	4.50 (4.50)	3.22 (3.34)	0.92 (0.94)
2	Ziegelstein (Brick)	Human Ratings	1.00	5.00	1.90	0.96
		CLAUS	1.74 (1.79)	3.82 (4.08)	2.94 (2.67)	0.29 (0.45)
		OCSAI	1.00 (1.00)	3.30 (3.00)	1.79 (1.54)	0.58 (0.57)
		GPT-4	1.00 (1.00)	4.50 (4.50)	2.29 (2.18)	1.02 (0.96)
3	Glühbirne (Light Bulb)	Human Ratings	1.00	5.00	2.35	1.07
		CLAUS	1.78 (1.84)	3.89 (3.96)	3.01 (2.91)	0.39 (0.40)
		OCSAI	1.00 (1.00)	3.70 (4.00)	1.97 (2.48)	0.69 (0.73)

		GPT-4	1.00 (1.00)	4.50 (4.50)	2.40 (2.44)	1.15 (1.18)
3	Holzlatte (Wooden Slat)	Human Ratings	1.00	5.00	2.89	1.03
		CLAUS	2.11 (2.13)	3.65 (3.92)	3.01 (3.10)	0.26 (0.23)
		OCSAI	1.00 (1.00)	3.70 (3.70)	2.08 (2.34)	0.43 (0.61)
		GPT-4	1.00 (1.00)	4.50 (4.50)	2.39 (2.32)	0.73 (0.84)
3	Löffel (Spoon)	Human Ratings	1.00	5.00	2.63	1.19
		CLAUS	2.15 (1.76)	4.01 (4.01)	2.98 (2.87)	0.36 (0.49)
		OCSAI	1.00 (1.00)	4.00 (4.80)	2.21 (2.82)	0.70 (0.72)
		GPT-4	1.00 (1.00)	4.50 (4.50)	2.73 (2.66)	1.16 (1.12)
3	Socken (Socks)	Human Ratings	1.00	5.00	2.60	1.16
		CLAUS	2.20 (1.88)	3.96 (3.97)	3.02 (3.05)	0.29 (0.33)
		OCSAI	1.00 (1.00)	4.30 (4.80)	2.44 (3.04)	0.67 (0.62)
		GPT-4	1.00 (1.00)	4.50 (4.50)	3.13 (3.20)	1.02 (1.03)
4	Glühbirne (Light Bulb)	Human Ratings	1.00	5.00	2.40	1.08
		CLAUS	1.78 (1.88)	3.92 (4.03)	2.98 (2.90)	0.40 (0.43)
		OCSAI	1.00 (1.00)	3.70 (4.40)	1.99 (2.52)	0.70 (0.74)
		GPT-4	1.00 (1.00)	4.50 (4.50)	2.42 (2.47)	1.18 (1.19)
4	Holzlatte (Wooden Slat)	Human Ratings	1.00	5.00	2.81	1.00
		CLAUS	2.10 (2.09)	3.67 (3.89)	2.99 (3.08)	0.26 (0.23)
		OCSAI	1.00 (1.00)	3.30 (3.70)	2.03 (2.26)	0.44 (0.60)
		GPT-4	1.00 (1.00)	4.50 (4.50)	2.35 (2.27)	0.71 (0.80)
4	Löffel (Spoon)	Human Ratings	1.00	5.00	2.66	1.22
		CLAUS	2.17 (1.77)	4.14 (3.99)	2.98 (2.89)	0.37 (0.50)
		OCSAI	1.00 (1.00)	3.70 (4.40)	2.18 (2.79)	0.69 (0.74)
		GPT-4	1.00 (1.00)	4.50 (4.50)	2.72 (2.67)	1.17 (1.15)
4	Socken (Socks)	Human Ratings	1.00	5.00	2.46	1.18
		CLAUS	2.04 (1.95)	3.91 (4.10)	2.99 (3.01)	0.31 (0.32)
		OCSAI	1.00 (1.00)	4.00 (4.40)	2.41 (3.05)	0.67 (0.63)
		GPT-4	1.00 (1.00)	4.50 (4.50)	3.10 (3.18)	1.02 (0.99)
5	Eimer (Bucket)	Human Ratings	1.17	4.83	2.73	0.60

		CLAUS	2.10 (1.81)	4.14 (4.04)	3.12 (3.03)	0.32 (0.33)
		OCSAI	1.00 (1.00)	4.30 (4.80)	2.40 (2.71)	0.54 (0.74)
		GPT-4	1.00 (1.00)	4.50 (4.50)	3.23 (2.90)	0.81 (0.94)
5	Gürtel (Belt)	Human Ratings	1.00	4.83	2.81	0.52
		CLAUS	1.85 (2.16)	4.03 (3.99)	3.14 (3.07)	0.30 (0.28)
		OCSAI	1.00 (1.00)	4.30 (5.00)	2.46 (2.62)	0.52 (0.78)
		GPT-4	1.00 (1.00)	4.50 (4.50)	3.39 (3.22)	0.76 (0.87)
5	Kerze (Candle)	Human Ratings	1.00	4.67	2.82	0.68
		CLAUS	2.25 (1.86)	4.29 (4.04)	3.30 (3.09)	0.31 (0.38)
		OCSAI	1.00 (1.00)	4.30 (4.80)	2.55 (2.96)	0.55 (0.70)
		GPT-4	1.00 (1.00)	4.50 (4.50)	3.21 (3.17)	0.93 (0.98)
5	Messer (Knife)	Human Ratings	1.00	4.67	2.72	0.65
		CLAUS	2.00 (2.08)	4.08 (4.27)	3.10 (3.10)	0.32 (0.31)
		OCSAI	1.00 (1.00)	4.70 (5.00)	2.45 (1.84)	0.50 (0.62)
		GPT-4	1.00 (1.00)	4.50 (4.50)	2.96 (2.83)	0.90 (1.01)
5	Socke (Sock)	Human Ratings	1.00	5.00	2.86	0.50
		CLAUS	2.06 (1.94)	4.15 (4.26)	3.25 (3.13)	0.27 (0.30)
		OCSAI	1.00 (1.00)	4.70 (4.80)	2.78 (3.36)	0.45 (0.54)
		GPT-4	1.00 (1.00)	4.50 (4.50)	3.51 (3.36)	0.64 (0.77)
6	Autoreifen (Car Tires)	Human Ratings	1.00	4.40	2.60	0.57
		CLAUS	1.68 (1.76)	4.06 (4.04)	3.10 (3.05)	0.30 (0.36)
		OCSAI	1.00 (1.00)	3.70 (4.50)	2.45 (3.16)	0.51 (0.78)
		GPT-4	1.00 (1.00)	4.50 (4.50)	3.33 (3.36)	0.87 (0.98)
6	Konservendose (Tin Can)	Human Ratings	1.00	4.67	2.47	0.52
		CLAUS	1.81 (1.87)	4.08 (4.22)	3.04 (2.99)	0.31 (0.35)
		OCSAI	1.00 (1.00)	4.00 (4.50)	2.38 (2.53)	0.51 (0.84)
		GPT-4	1.00 (1.00)	4.50 (4.50)	3.14 (2.76)	0.89 (0.97)
6	Stift (Pen)	Human Ratings	1.00	4.67	2.34	0.67
		CLAUS	2.07 (1.80)	4.16 (4.04)	3.02 (2.86)	0.36 (0.50)
		OCSAI	1.00 (1.00)	4.30 (4.80)	2.32 (2.76)	0.79 (0.89)

		GPT-4	1.00 (1.00)	4.50 (4.50)	2.68 (2.52)	1.11 (1.07)
6	Ziegelstein (Brick)	Human Ratings	1.00	4.50	2.53	0.68
		CLAUS	1.74 (1.73)	4.26 (4.26)	3.07 (2.83)	0.27 (0.44)
		OCSAI	1.00 (1.00)	3.70 (4.00)	2.10 (1.71)	0.59 (0.58)
		GPT-4	1.00 (1.00)	4.50 (4.50)	2.73 (2.53)	1.00 (0.99)
7	Autoreifen (Car Tires)	Human Ratings	1.00	4.83	2.43	0.70
		CLAUS	1.63 (1.81)	4.05 (4.08)	3.14 (3.08)	0.28 (0.32)
		OCSAI	1.00 (1.00)	4.30 (4.50)	2.52 (3.24)	0.49 (0.70)
		GPT-4	1.00 (1.00)	4.50 (4.50)	3.40 (3.40)	0.79 (0.93)
7	Stift (Pen)	Human Ratings	1.00	4.50	2.51	0.74
		CLAUS	2.10 (1.80)	4.09 (4.03)	3.11 (3.02)	0.33 (0.44)
		OCSAI	1.00 (1.00)	4.70 (5.00)	2.58 (3.04)	0.70 (0.77)
		GPT-4	1.00 (1.00)	4.50 (4.50)	2.98 (2.85)	1.00 (0.99)
7	Ziegelstein (Brick)	Human Ratings	1.00	4.50	2.36	0.59
		CLAUS	2.05 (1.77)	4.04 (4.18)	3.12 (2.91)	0.27 (0.42)
		OCSAI	1.00 (1.00)	4.00 (4.00)	2.21 (1.82)	0.56 (0.57)
		GPT-4	1.00 (1.00)	4.50 (4.50)	2.95 (2.73)	0.96 (0.99)
8	Messer (Knife)	Human Ratings	1.00	5.00	2.51	0.93
		CLAUS	2.05 (2.17)	4.18 (4.02)	3.03 (3.04)	0.31 (0.31)
		OCSAI	1.00 (1.00)	4.00 (4.00)	2.35 (1.76)	0.54 (0.63)
		GPT-4	1.00 (1.00)	4.50 (4.50)	2.85 (2.69)	1.07 (1.13)

Table S6. *Rater statistics including Large Language Models.*

Study	Rater/LLM	α	α if Rater is deleted	Rater Total Correlation
1	Human Rater 1	.724	.684	.717
	Human Rater 2		.620	.784
	Human Rater 3		.586	.817
	CLAUS		.745	.641
	Human Rater 1	.732	.672	.744
	Human Rater 2		.657	.760
	Human Rater 3		.599	.818
	CLAUS (Translation)		.745	.655
	Human Rater 1	.788	.768	.740
	Human Rater 2		.725	.795
	Human Rater 3		.702	.822
	OCSAI		.745	.770
	Human Rater 1	.755	.723	.729
	Human Rater 2		.663	.798
	Human Rater 3		.652	.809
	OCSAI (Translation)		.745	.701
	Human Rater 1	.782	.758	.741
	Human Rater 2		.719	.791
	Human Rater 3		.691	.822
	GPT-4		.745	.758
	Human Rater 1	.724	.730	.735
	Human Rater 2		.683	.791
	Human Rater 3		.661	.815
	GPT-4 (Translation)		.745	.716
5	Human Rater 1	.832	.797	.760
	Human Rater 2		.803	.735
	Human Rater 3		.808	.709
	Human Rater 4		.801	.744
	Human Rater 5		.823	.638
	Human Rater 6		.803	.736
	CLAUS		.827	.616
	Human Rater 1	.833	.798	.763
	Human Rater 2		.804	.735
	Human Rater 3		.809	.713
	Human Rater 4		.802	.743
	Human Rater 5		.825	.631
	Human Rater 6		.804	.737
	CLAUS (Translation)		.827	.622
	Human Rater 1	.852	.824	.767
	Human Rater 2		.828	.746
	Human Rater 3		.835	.707
	Human Rater 4		.827	.751
	Human Rater 5		.849	.631

	Human Rater 6		.829	.741
	OCSAI		.827	.750
	Human Rater 1	.839	.805	.771
	Human Rater 2		.813	.732
	Human Rater 3		.817	.712
	Human Rater 4		.810	.746
	Human Rater 5		.835	.619
	Human Rater 6		.809	.749
	OCSAI (Translation)		.827	.662
	Human Rater 1	.841	.807	.779
	Human Rater 2		.818	.725
	Human Rater 3		.818	.726
	Human Rater 4		.814	.746
	Human Rater 5		.840	.611
	Human Rater 6		.814	.745
	GPT-4		.827	.680
	Human Rater 1	.832	.807	.772
	Human Rater 2		.815	.736
	Human Rater 3		.818	.718
	Human Rater 4		.812	.748
	Human Rater 5		.838	.618
	Human Rater 6		.814	.738
	GPT-4 (Translation)		.827	.676
6	Human Rater 1	.834	.803	.750
	Human Rater 2		.839	.571
	Human Rater 3		.810	.719
	Human Rater 4		.799	.766
	Human Rater 5		.817	.688
	Human Rater 6		.792	.801
	CLAUS		.817	.689
	Human Rater 1	.847	.819	.760
	Human Rater 2		.852	.588
	Human Rater 3		.829	.711
	Human Rater 4		.817	.769
	Human Rater 5		.836	.677
	Human Rater 6		.810	.805
	CLAUS (Translation)		.817	.767
	Human Rater 1	.856	.830	.765
	Human Rater 2		.862	.588
	Human Rater 3		.840	.711
	Human Rater 4		.830	.768
	Human Rater 5		.846	.684
	Human Rater 6		.822	.805
	OCSAI		.817	.831
	Human Rater 1	.834	.803	.748
	Human Rater 2		.833	.602
	Human Rater 3		.812	.705
	Human Rater 4		.800	.762

	Human Rater 5		.819	.671
	Human Rater 6		.790	.806
	OCSAI (Translation)		.817	.682
	Human Rater 1	.849	.824	.750
	Human Rater 2		.853	.593
	Human Rater 3		.831	.711
	Human Rater 4		.819	.773
	Human Rater 5		.840	.668
	Human Rater 6		.810	.817
	GPT-4		.817	.781
	Human Rater 1	.834	.819	.744
	Human Rater 2		.848	.591
	Human Rater 3		.824	.714
	Human Rater 4		.813	.771
	Human Rater 5		.833	.668
	Human Rater 6		.802	.818
	GPT-4 (Translation)		.817	.746
7	Human Rater 1	.822	.782	.767
	Human Rater 2		.803	.673
	Human Rater 3		.807	.652
	Human Rater 4		.792	.725
	Human Rater 5		.799	.694
	Human Rater 6		.787	.744
	CLAUS		.816	.612
	Human Rater 1	.840	.808	.765
	Human Rater 2		.827	.668
	Human Rater 3		.827	.667
	Human Rater 4		.813	.737
	Human Rater 5		.824	.683
	Human Rater 6		.810	.754
	CLAUS (Translation)		.816	.626
	Human Rater 1	.850	.821	.769
	Human Rater 2		.841	.664
	Human Rater 3		.840	.670
	Human Rater 4		.826	.747
	Human Rater 5		.839	.677
	Human Rater 6		.824	.757
	OCSAI		.816	.797
	Human Rater 1	.827	.793	.749
	Human Rater 2		.818	.635
	Human Rater 3		.804	.701
	Human Rater 4		.792	.757
	Human Rater 5		.816	.643
	Human Rater 6		.788	.773
	OCSAI (Translation)		.816	.645
	Human Rater 1	.830	.797	.753
	Human Rater 2		.811	.686
	Human Rater 3		.816	.661

	Human Rater 4		.802	.729
	Human Rater 5		.812	.680
	Human Rater 6		.796	.755
	GPT-4		.816	.671
	Human Rater 1	.822	.790	.756
	Human Rater 2		.808	.674
	Human Rater 3		.810	.665
	Human Rater 4		.795	.732
	Human Rater 5		.808	.673
	Human Rater 6		.789	.759
	GPT-4 (Translation)		.816	.638
8	Human Rater 1	.879	.816	.900
	Human Rater 2		.828	.884
	Human Rater 3		.827	.885
	CLAUS		.903	.760
	Human Rater 1	.892	.842	.899
	Human Rater 2		.848	.889
	Human Rater 3		.846	.892
	CLAUS (Translation)		.903	.797
	Human Rater 1	.916	.886	.903
	Human Rater 2		.887	.902
	Human Rater 3		.889	.898
	OCSAI		.903	.873
	Human Rater 1	.904	.870	.891
	Human Rater 2		.863	.902
	Human Rater 3		.866	.898
	OCSAI (Translation)		.903	.835
	Human Rater 1	.894	.848	.896
	Human Rater 2		.845	.900
	Human Rater 3		.855	.886
	GPT-4		.903	.805
	Human Rater 1	.879	.848	.896
	Human Rater 2		.845	.900
	Human Rater 3		.854	.886
	GPT-4 (Translation)		.903	.804

Notes. Each section should be interpreted separately per study.

All values were z-transformed.
